# A Novel Heterostructure of BiOI Nanosheets Anchored onto MWCNTs with Excellent Visible-Light Photocatalytic Activity

**DOI:** 10.3390/nano7010022

**Published:** 2017-01-23

**Authors:** Shijie Li, Shiwei Hu, Kaibing Xu, Wei Jiang, Jianshe Liu, Zhaohui Wang

**Affiliations:** 1 Innovation & Application Institute, Zhejiang Ocean University, Zhoushan 316022, China; hushiweihai@163.com (S.H.); jiangwei19871216@163.com (W.J.); 2 Zhejiang Provincial Key Laboratory of Health Risk Factors for Seafood, Zhoushan Municipal Center for Disease Control and Prevention, Zhoushan 316021, China; 3 State Key Laboratory for Modification of Chemical Fibers and Polymer Materials, Research Center for Analysis and Measurement, Donghua University, Shanghai 201620, China; xukaibing@dhu.edu.cn; 4 State Environmental Protection Engineering Center for Pollution Treatment and Control in Textile Industry, College of Environmental Science and Engineering, Donghua University, Shanghai 201620, China; liujianshe@dhu.edu.cn; 5 International Centre for Balanced Land Use (ICBLU), the University of Newcastle, Callaghan, NSW 2308, Australia

**Keywords:** MWCNTs/BiOI, nanocomposites, photocatalysis, visible-light

## Abstract

Developing efficient visible-light-driven (VLD) photocatalysts for environmental decontamination has drawn significant attention in recent years. Herein, we have reported a novel heterostructure of multiwalled carbon nanotubes (MWCNTs) coated with BiOI nanosheets as an efficient VLD photocatalyst, which was prepared via a simple solvothermal method. The morphology and structure were characterized by powder X-ray diffraction (XRD), scanning electron microscopy (SEM), transmission electron microscopy (TEM), UV-Vis diffuse reflectance spectroscopy (DRS), and specific surface area measurements. The results showed that BiOI nanosheets were well deposited on MWCNTs. The MWCNTs/BiOI composites exhibited remarkably enhanced photocatalytic activity for the degradation of rhodamine B (RhB), methyl orange (MO), and *para*-chlorophenol (4-CP) under visible-light, compared with pure BiOI. When the MWCNTs content is 3 wt %, the MWCNTs/BiOI composite (3%M-Bi) achieves the highest activity, which is even higher than that of a mechanical mixture (3 wt % MWCNTs + 97 wt % BiOI). The superior photocatalytic activity is predominantly due to the strong coupling interface between MWCNTs and BiOI, which significantly promotes the efficient electron-hole separation. The photo-induced holes (h^+^) and superoxide radicals (O_2_^−^) mainly contribute to the photocatalytic degradation of RhB over 3%M-Bi. Therefore, the MWCNTs/BiOI composite is expected to be an efficient VLD photocatalyst for environmental purification.

## 1. Introduction

Semiconductor photocatalysis is an effective means to tackle the energy crisis and environmental problems by splitting water to produce H_2_ and degrading various organic contaminants in water/air [1]. The key aspect of the photocatalysis technique is the development of efficient and stable photocatalysts [2]. To effectively utilize solar energy, a variety of visible-light-driven (VLD) photocatalysts have been explored, including simple oxides (e.g., Ag_2_O [3], CuO [4]), complex oxides (e.g., Bi_2_Mo(W)O_6_ [5,6], BiOBr(Cl/I) [7,8,9], Bi_4_O_5_I_2_ [10]), sulfides (e.g., MoS_2_ [11], WS_2_ [12]), and nitrides (e.g., Ta_3_N_5_ [13], C_3_N_4_ [14]). However, most of the single-component VLD photocatalysts suffer from a fast recombination rate of photogenerated electron-hole pairs and a narrow photo-response range [15,16]. 

The construction of semiconductor composites can optimize the capture of light and promote the separation of photo-induced charge, thus leading to an enhanced photocatalytic activity [17,18,19]. BiOI (band gap: 1.77–1.92 eV) has been put forward as one of the most promising VLD photocatalysts because the layer structure composed of [Bi_2_O_2_]^2+^ slabs interleaved by double slabs of I atoms can facilitate the separation of electron-hole pairs. To further improve its photocatalytic activity, many kinds of BiOI-based composites have been developed, including BiOI-oxide (oxide: Fe_2_O_3_ [20], TiO_2_ [21,22], WO_3_ [23] BiOBr(Cl) [24,25], BiVO_4_ [26], Bi_12_O_17_Cl_2_ [27], Bi_2_W(Mo)O_6_ [28,29], BiOIO_3_ [30], Bi_4_Ti_3_O_12_ [31], BiPO_4_ [32]), BiOI-sulfide (sulfide: CdS [33], Bi_2_S_3_ [34]), BiOI-metal (metal: Ag [35]), BiOI-carbon (carbon: graphene [36,37,38,39], carbon quantum dots [40]), and multi-component (e.g., BiOClx/BiOBry/BiOIz [41], MoS_2_/AgI/BiOI [42], Ag_3_VO_4_/Ag/BiOI [43]). These composites all show relatively higher activity than pure BiOI in degrading organic contaminants, Cr(VI) reduction, and/or hydrogen production. The BiOI-carbon composites have particularly been demonstrated to be excellent photocatalysts, but they are still underdeveloped [36,37,38,39,40].

Carbon nanotubes (CNTs) have been recognized as excellent electron-acceptor/transport matrix in photocatalysis due to their unique structure and excellent electronic properties. Therefore, various CNTs-based composites [44,45,46,47,48] (e.g., CNTs/TiO_2_, CNTs/Bi_2_O_2_CO_3_, CNTs/Bi_2_WO_6_) have been developed and have demonstrated better photocatalytic activity. Thus, it is a promising alternative to construct multiwalled carbon nanotubes (MWCNTs)/BiOI composites with synergistic effects for enhancing photocatalytic activity [49]. However, to the best of our knowledge, photocatalysis using the novel heterostructure of BiOI nanosheets anchored onto MWCNTs has not been reported. 

In the present work, the novel solvothermally prepared MWCNTs/BiOI heterostructure showed markedly higher activity than pure BiOI. The best photocatalytic activity was achieved with the proportion of 3 wt % MWCNTs in the composite. A possible mechanism for the enhanced visible-light photocatalytic activity of MWCNTs/BiOI is proposed.

## 2. Results and Discussion

### 2.1. Characterization of Photocatalysts

**Figure 1:**
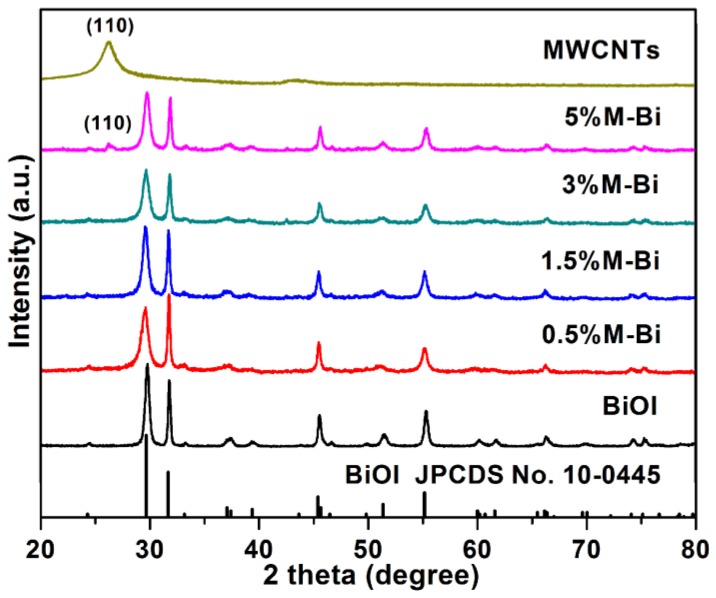
X-ray diffraction (XRD) patterns of as-prepared multiwalled carbon nanotubes (MWCNTs)/BiOI composites, BiOI, and MWCNTs.

Figure 1 presents the XRD patterns of the as-prepared MWCNTs/BiOI composites, BiOI, and MWCNTs. All diffraction peaks from BiOI can be readily indexed to tetragonal BiOI (JCPDS No. 10-0445). The broad peak at 2θ = 26.3° is assigned to the characteristic peak of MWCNTs [47,48]. For MWCNTs/BiOI composites, when the MWCNTs content is low (0.5, 1.5, or 3 wt %), the XRD patterns of MWCNTs/BiOI composites exhibit BiOI peaks. Six characteristic peaks at 2θ = 29.7°, 2θ = 31.6°, 2θ = 37.1°, 2θ = 39.4°, 2θ = 45.3°, and 2θ = 55.2° are indexed to the (102), (110), (103), (004), (200) and (212) planes of tetragonal BiOI, respectively. When the MWCNTs content is increased to 5 wt %, the characteristic peak of MWCNTs at 2θ = 26.3° appears, which corresponds to the (110) plane of MWCNTs, verifying the formation of MWCNTs/BiOI composites. In addition, no impurity phase is detected, suggesting the high purity of these composites.

**Figure 2:**
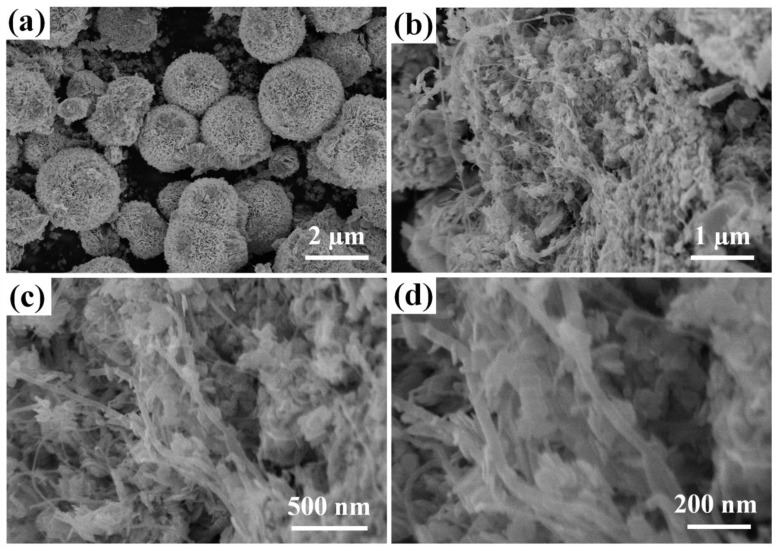
Scanning electron microscopy (SEM) images of (**a**) BiOI, and (**b**–**d**) 3%M-Bi.

The morphologies of BiOI and the MWCNTs/BiOI composite (3%M-Bi) were studied by SEM. Pristine BiOI presents flower-like microspheres (diameters: 1–3.5 μm, Figure 2a) comprised of two-dimensional (2D) nanosheets (thickness: 15 nm, Supplementary Material Figure S1). In contrast, 3%M-Bi presents a one-dimensional shape, where BiOI nanosheets (thickness: 15 nm, diameter: 20–100 nm) were uniformly anchored onto the MWCNTs backbone (Figure 2b–d). 

**Figure 3:**
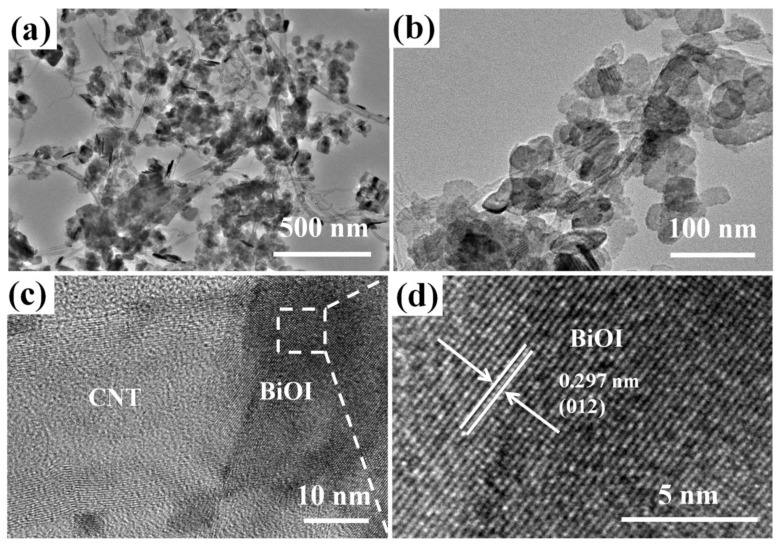
(**a**–**d**) Transmission electron microscopy (TEM) images of 3%M-Bi.

Further information about 3%M-Bi is obtained from TEM and high-resolution transmission electron microscopy (HRTEM) measurements (Figure 3). The TEM images further confirm that BiOI nanosheets are tightly deposited on the surface of MWCNTs (Figure 3a,b). Moreover, the lattices of BiOI and MWCNTs intersect each other (Figure 3c). The lattice fringe with an inter-planar distance of 0.297 nm coincides with the (012) crystal plane of BiOI (Figure 3d). These facts confirm the formation of MWCNTs/BiOI with intimate contact between MWCNTs and BiOI.

**Figure 4:**
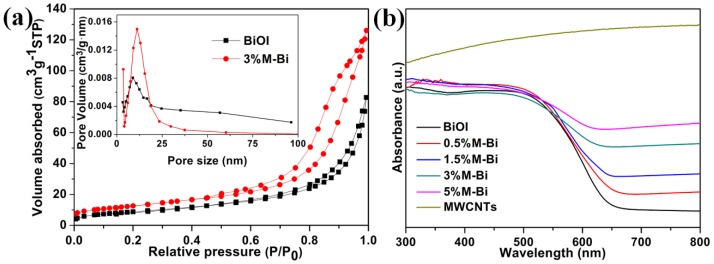
(**a**) N_2_ adsorption/desorption isotherms of BiOI and 3%M-Bi; the inset is the corresponding pore size distributions; (**b**) UV-Vis reflectance spectra of BiOI, MWCNTs, and MWCNTs/BiOI composites.

The N_2_ adsorption/desorption isotherms of BiOI and 3%M-Bi were measured (Figure 4a). The Brunauer-Emmett-Teller (BET) specific surface area of BiOI is determined to be 25.7 m^2^·g^−1^. Interestingly, 3%M-Bi displays an apparent increase of the BET surface area to 44.5 m^2^·g^−1^. The high surface area is primarily caused by the MWCNTs, which could offer more adsorptive and reactive sites for organic pollutants. The pore size distributions, which are calculated from the desorption branches, indicate the existence of nano-pores with diameters of ~9 and 11 nm for BiOI and 3%M-Bi, respectively. Significantly, the presence of nano-pores is favorable for photocatalysis [50]. 

The absorption spectra of BiOI, MWCNTs, and MWCNTs/BiOI composites were recorded (Figure 4b). BiOI displays strong absorption from the ultraviolet (UV) to visible light (VL) region with an absorption edge located at ~650 nm (band gap: 1.9 eV), consistent with previous reports [35,43]. MWCNTs exhibit excellent light absorption in the whole wavelength range tested, in agreement with the previous observation [48]. The incorporation of MWCNTs broadens and enhances the VL absorption of BiOI, and the wavelength thresholds of MWCNTs/BiOI composites are determined to be 680–851 nm, corresponding to the band gap of 1.82–1.46 eV, which favors the efficient utilization of solar energy.

### 2.2. Photocatalytic Performances

**Figure 5:**
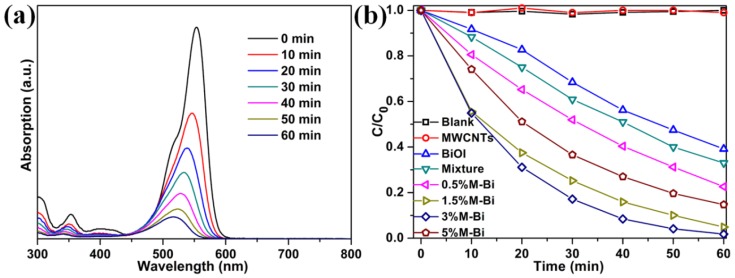
(**a**) UV-Vis absorption spectra of rhodamine B (RhB) dye versus reaction time over 3%M-Bi; (**b**) Photocatalytic degradation of RhB under visible light (λ > 400 nm), in the absence of catalysts and in the presence of as-prepared catalysts (10 mg).

The photocatalytic performance of MWCNTs/BiOI composites in degrading cationic dye rhodamine B (RhB) under visible light (λ > 400 nm) was studied (Figure 5). Figure 5a presents the temporal evolution of the absorption spectra of RhB with 3%M-Bi as a photocatalyst; the characteristic absorption peak of RhB at 554 nm diminishes rapidly as the irradiation time increases (Figure 5a). After 60 min of reaction, the RhB photodegradation efficiency reaches 98.3% (Figure 5b). For comparison, nearly no RhB is degraded without photocatalyts and with MWCNTs as the photocatalyst after 60 min of visible light, and 50.9%, 77.4%, 95.1%, or 85.3% of RhB is degraded after 60 min of reaction when using BiOI, 0.5%M-Bi, 1.5%M-Bi, or 5%M-Bi as the photocatalyst, respectively. Obviously, all MWCNTs/BiOI composites display significantly enhanced activity compared with pristine BiOI, mainly resulting from the synergistic effect between the MWCNTs and BiOI. Among these composites, 3%M-Bi achieves the highest photocatalytic activity, indicating the optimum content of MWCNTs is ~3 wt%. To further analyze the role of the heterojunction in the MWCNTs/BiOI composite, the RhB degradation over a mechanical mixture (97% BiOI + 3%MWCNTs) was further studied. The degradation efficiency (66.9%) is much lower than that (98.3%) achieved by 3%M-Bi, demonstrating that the strong coupling interface between MWCNTs and BiOI is crucial for the improvement of photocatalytic activity.

In addition, the photocatalytic degradation of RhB over TiO_2_ (P25) or the catalysts (including CNTs/Bi_2_O_2_CO_3_ [45], Ag_2_O/BiOCOOH [51], and Ta_3_N_5_-Pt [50]) reported by our group were investigated (Figure S2), and the degradation efficiencies was 15.1%, 32.3%, 77.9%, or 100% after 60 min of reaction, respectively. Obviously, 3%M-Bi was much more active than P25, CNTs/Bi_2_O_2_CO_3_, and Ag_2_O/BiOCOOH, demonstrating that 3%M-Bi displayed excellent photocatalytic activity.

The photocatalytic degradation of RhB followed the pseudo-first-order model [51]: −ln(*C*/*C*_0_) = *kt*, where *C*_0_ and *C* are the original concentration of RhB and the concentration of RhB at time *t*, respectively. The *k* is the reaction rate constant. The *k* values along with the correlation factors (*R*^2^), are shown in Figure S3. The *k* values of MWCNTs/BiOI composites (0.02441 min^−1^ for 0.5%M-Bi, 0.0475 min^−1^ for 1.5%M-Bi, 0.0663 min^−1^ for 3%M-Bi, and 0.03228 min^−1^ for 5%M-Bi) are apparently higher than those of MWCNTs (~0 min^−1^), BiOI (0.01613 min^−1^), and a mechanical mixture of MWCNTs and BiOI (0.01891 min^−1^) (Figure S3). Notably, 3%M-Bi has the highest photodegradation rate constant (0.0663 min^−1^).

Moreover, the photocatalytic degradation of anionic dye methyl orange (MO) with as-prepared catalysts was also studied (Figure S4). 3%M-Bi still achieves the highest photodegradation efficiency (84.2%) and rate constant (0.01022 min^−1^) after 3 h of reaction. This fact indicates that 3%M-Bi can efficiently decompose anionic dye MO as well.

**Figure 6:**
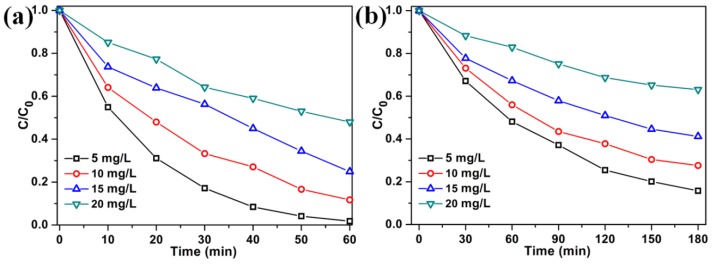
The effect of the initial concentrations of RhB (**a**) and methyl orange (MO) (**b**) on the photocatalytic activity of 3%M-Bi (10 mg).

The effect of the initial concentrations of RhB or MO on the photocatalytic activity of 3%M-Bi was investigated (Figure 6). When the initial concentration of RhB or MO is elevated from 5 mg·L^−1^ to 20 mg·L^−1^, the degradation efficiency of RhB or MO decreases from 98.3% or 84.2% to 51.7% or 36.7%, respectively. On the one hand, when the RhB or MO concentration is high, a large portion of visible-light is absorbed by RhB or MO rather than by 3%M-Bi, resulting in lower degradation efficiency. On the other hand, the intermediates produced during the reaction occupy part of the limited adsorptive and catalytic sites on 3%M-Bi, which could inhibit RhB or MO degradation. Such suppression will be more significant in the presence of a rising level of intermediates generated upon an increased initial dye concentration [51,52].

**Figure 7:**
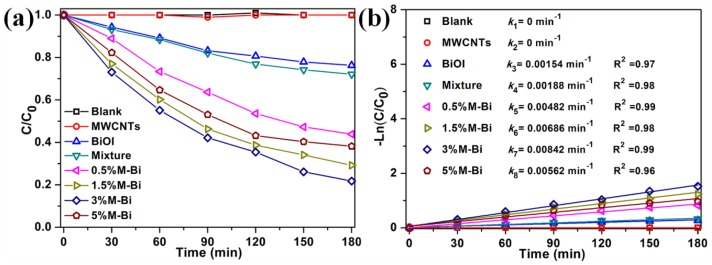
The degradation efficiencies (**a**) and rate constants (**b**) of *para*-chlorophenol (4-CP) in aqueous solution (1 mg·L^−1^, 50 mL) versus the exposure time under visible light (λ > 400 nm), in the absence of catalysts and in the presence of as-prepared catalysts (10 mg).

To further confirm that the photocatalytic activity of MWCNTs/BiOI composites originates from the excitation of the catalysts rather than the dye sensitization mechanism, the photocatalytic degradation of colorless neutral *para*-chlorophenol (4-CP) over MWCNTs/BiOI composites was also performed (Figure 7). The degradation of 4-CP is extremely slow without photocatalysts and with MWCNTs after 180 min of reaction. The degradation efficiency approaches 56.1%, 70.8%, 78.3%, or 61.8% by using 0.5%M-Bi, 1.5%M-Bi, 3%M-Bi, or 5%M-Bi as the photocatalyst, respectively. Among these composites, 3%M-Bi exhibits the highest photodegradation efficiency (78.3%), which is much higher than those from BiOI (23.7%), and a mixture of MWCNTs and BiOI (27.9%). The photodegradation rate constant using 3%M-Bi (0.00842 min^−1^) is about 4.47 or 3.48 times higher than that using BiOI (0.00154 min^−1^), or a mixture of the two components (0.00188 min^−1^). 

From the above results, one can conclude that 3%M-Bi can efficiently degrade different kinds of organic pollutants (such as cationic RhB, anionic MO, and neutral 4-CP). The incorporation of a suitable amount of MWCNTs can dramatically improve the activity of BiOI, due to the synergic effect between MWCNTs and BiOI.

**Figure 8:**
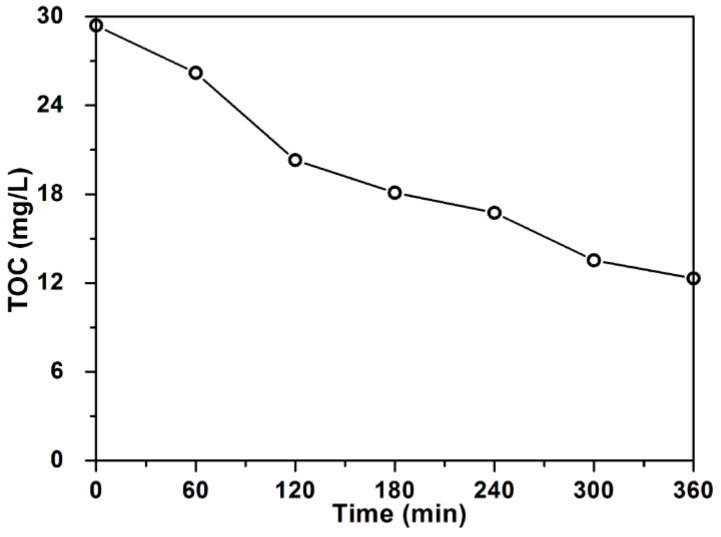
Total organic carbon (TOC) removal during RhB degradation (40 mg·L^−1^, 100 mL) over 3%M-Bi (100 mg) under visible light (λ > 400 nm).

The total organic carbon (TOC) value is a significant index for the mineralization degree of organic species. Herein, the mineralization of RhB was studied by using 3%M-Bi as the catalyst (Figure 8). As the irradiation time increases, the TOC value continuously declines from 29.4 mg·L^−1^ at 0 h to 12.31 mg·L^−1^ at 6 h, achieving a high mineralization ratio of 58.2%. This fact confirms that 3%M-Bi can effectively mineralize RhB dye under visible light.

**Figure 9:**
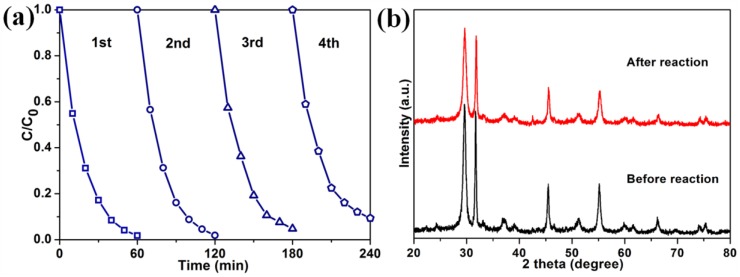
(**a**) Cycling runs in photocatalytic degradation of RhB (5 mg·L^−1^, 50 mL) over 3%M-Bi (10 mg) under visible light (λ > 400 nm); (**b**) XRD patterns of 3%M-Bi before and after the reaction.

The stability of a catalyst is highly important for its practical application. Thus, four successive runs in RhB degradation over 3%M-Bi were performed. After four runs, 3%M-Bi still has a high activity with RhB degradation efficiency of 90.6% (Figure 9a). XRD characterization demonstrates that the used 3%M-Bi presents similar diffraction peaks as the fresh one (Figure 9b). These facts indicate that 3%M-Bi has good stability.

**Figure 10:**
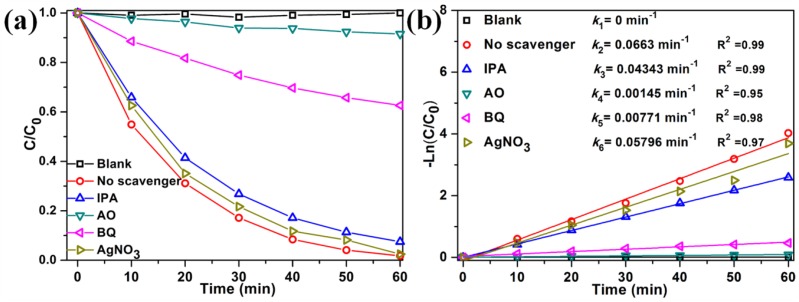
Effect of different scavengers on the degradation efficiencies (**a**) and rate constants (**b**) of RhB over 3%M-Bi under visible light (λ > 400 nm).

To analyze the photocatalytic reaction mechanism, it is of vital importance to identify the primary active species. Thus, the radical trapping experiments were performed (Figure 10). Scavengers such as benzoquinone (BQ), ammonium oxalate (AO), silver nitrate (AgNO_3_), and isopropyl alcohol (IPA) are commonly used to capture superoxide radical anions (O_2_·^−^), photogenerated holes (h^+^), electrons (e^−^), and hydroxyl free radicals (•OH), respectively [51]. The photocatalytic activity of 3%M-Bi is not markedly influenced by the addition of AgNO_3_ or IPA, indicating that •OH and photogenerated e^−^ are not the primary active species. However, the activity is greatly inhibited by the addition of BQ or AO. After 60 min of reaction, the photodegradation efficiency decreases from 98.3% to 37.4% or 8.5%, respectively (Figure 10a). The photocatalytic degradation process matched well with the pseudo-first-order model. Apparently, the rate constant also declined from 0.0663% to 0.00771% or 0.00145% in the presence of BQ or AO, respectively (Figure 10b). These facts reveal that O_2_·^−^ and photogenerated h^+^ mainly contribute to the RhB degradation. 

**Figure 11:**
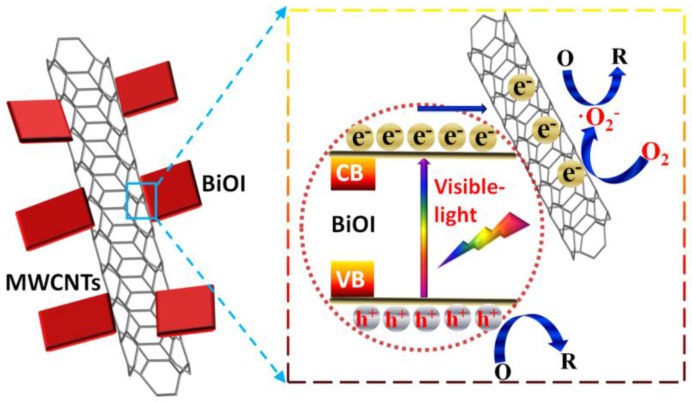
The proposed photocatalytic mechanism for the high activity of MWCNTs/BiOI.

Based on the experimental results and band edge positions (Figure 11), the excellent photocatalytic activity of MWCNTs/BiOI is attributed to the intimate contact between MWCNTs and BiOI nanosheets in this novel heterostructure. The CNTs/semiconductor composite is an effective architecture for efficient charge transfer and separation, leading to the high photocatalytic activity. The intimate attachment of MWCNTs with BiOI nanosheets in the composite would greatly promote the efficient separation of photogenerated electron–hole pairs [19,47]. Under visible light, the photogenerated e^−^ would transfer from the valence band (VB) to the conduction band (CB), leaving the h^+^ in the CB of BiOI. The photogenerated e^−^ quickly flows into MWCNTs, resulting in a low electron-hole recombination rate [19,47]. According to the results of radical trapping experiments (Figure 10), the h^+^ stored in the VB of BiOI can directly oxidize RhB/MO/4-CP. Simultaneously, the e^−^ accumulated on MWCNTs would reduce O_2_ to produce strong oxidizing species O_2_·^−^, which further degrade RhB/MO/4-CP. Consequently, the MWCNTs/BiOI composites exhibit dramatically enhanced photocatalytic activity. 

## 3. Materials and Methods

### 3.1. Materials

Bismuth nitrate pentahydrate (Bi(NO_3_)_3_·5H_2_O), potassium iodide (KI), ethylene glycol (EG), and absolute ethanol (CH_3_CH_2_OH) were purchased from Sinopharm Chemical Reagent Co., Ltd., Shanghai, China. MWCNTs were purchased from XFNANO, INC Advanced Materials Supplier (Nanjing, China).

### 3.2. Preparation of MWCNTs/BiOI Composites

The MWCNTs were purified according to a previous report [46]. Briefly, 2 g of MWCNTs and 300 mL of 65% HNO_3_ were added in a flask, and the mixture was refluxed for 2 h. After the system cooled down to room temperature, the product was filtered and washed with deionized water until the pH of the solution was 7. Then the collected product was dried at 90 °C overnight. MWCNTs/BiOI composites were prepared solvothermally. Typically, a desired amount of MWCNTs was ultrasonically dispersed in the mixture (30 mL ethylene glycol + 10 mL absolute ethanol). Subsequently, 1 mmol Bi(NO_3_)_3_·5H_2_O and 1 mmol KI were dissolved in the above solution with ultrasonication. The mixture was transferred to a 50 mL Teflon-lined autoclave and heated in an oven at 150 °C for 20 h. After cooling down naturally, the collected solids were washed with water-ethanol 5 times, and were then dried at 75 °C overnight in an oven. Finally, MWCNTs/BiOI composites with 0.5, 1.5, 3, and 5 wt % MWCNTs were obtained and defined as 0.5%M-Bi, 1%M-Bi, 3%M-Bi, and 5%M-Bi, respectively. 

### 3.3. Characterization

X-ray diffraction (XRD) measurements were recorded on a Rigaku D/max-2550 PC (Tokyo, Japan) X-ray diffractometer using Cu Ka radiation (λ = 0.15418 nm). Scanning electron microscope (SEM) tests were performed on a Hitachi S-4800 field emission scanning electron microscope (Tokyo, Japan). Transmission electron microscope (TEM) tests were conducted by a JEOL JEM-2100 high-resolution transmission electron microscope (Tokyo, Japan). N_2_ adsorption-desorption isotherms were determined by Brunauer-Emmett-Teller measurements (BET, Micromeritics ASAP 2020, Norcross, GA, USA). Optical diffuse reflectance spectra were obtained on a UV-Vis-NIR scanning spectrophotometer (Shimadzu UV-2600, Kokyo, Japan) with an integrating sphere accessory.

### 3.4. Photocatalytic Degradation Experiments

Photocatalytic performance of as-prepared catalysts was evaluated by degrading cationic dye rhodamine B (RhB), anionic dye methyl orange (MO), and neutral colorless *para*-chlorophenol (4-CP) using a xenon lamp (300 W, Perfect Light Co. Ltd. Beijing, China) with a cutoff filter (λ > 400 nm) as the visible-light source. Typically, 10 mg of photocatalyst was dispersed in 50 mL of RhB (5 mg·L^−1^), MO (5 mg·L^−1^), or 4-CP (1 mg·L^−1^) solution in a beaker. The solution was vigorously stirred for 0.5 h in the dark before the reaction. During the irradiation, 2 mL of solution was sampled at certain intervals and centrifuged for analysis. The RhB or MO concentrations in the solution were determined at 554 nm or 463 nm by a UV-2600 spectrophotometer. The 4-CP concentrations in the solution were analyzed by high-performance liquid chromatography (HPLC) using an Agilent 1100 series (Santa Clara, CA, USA) equipped with a UV detector at 280 nm. The mobile phase was composed of 80% methanol and 20% water at a flow rate of 0.5 mL·min^−1^.

The total organic carbon (TOC) test was performed by adding 100 mg 3%M-Bi into RhB aqueous solution (100 mL, 40 mg·L^−1^). The suspension was magnetically stirred for 60 min in the dark before the reaction. During the irradiation, 10 mL of the suspension was sampled at given time intervals (60 min), and filtered through a membrane (pore size: 0.45 μm). Subsequently, the resulting solution was examined by a Shimadzu TOC (Tokyo, Japan) analyzer. 

In the recycling test, four consecutive runs for RhB degradation over 3%M-Bi were carried out. After each run, the catalysts were collected by a simple precipitation procedure and washed thoroughly with water and dried, and were then dispersed in fresh RhB aqueous solution (50 mL, 5 mg·L^−1^) again.

Radical scavenging experiments were conducted by adding 6 mM silver nitrate (AgNO_3_), 1 mM ammonium oxalate (AO), 1 mM *p*-benzoquinone (BQ), or 1 mM iso-propanol (IPA) into the RhB (50 mL, 5 mg·L^−1^) solution before the photocatalytic tests.

## 4. Conclusions

A novel heterostructure of MWCNTs decorated with BiOI nanosheets was prepared by a facile one-pot solvothermal method. The MWCNTs/BiOI composite with 3 wt % MWCNTs achieved the highest photocatalytic activity, which is much higher than pristine BiOI, and a mechanical mixture of BiOI and MWCNTs. Moreover, it can efficiently degrade different kinds of organic pollutants (such as RhB/MO/4-CP) with excellent stability. The superior photocatalytic activity of MWCNTs/BiOI can be ascribed to the synergistic effect of MWCNTs and BiOI, facilitating the sufficient utilization of visible-light and the efficient separation of photogenerated charge carriers. Therefore, the MWCNTs/BiOI composite has great potential for environmental remediation applications.

## Supplementary Materials

The following are available online at http://www.mdpi.com/2079-4991/7/1/22/s1.
