# Supplementary material for: A Novel Heterostructure of BiOI Nanosheets Anchored onto MWCNTs with Excellent Visible-Light Photocatalytic Activity
Source: Nanomaterials (Basel). 2017 Jan 23;7(1):22. doi: 10.3390/nano7010022 (PMC5295212; doi:10.3390/nano7010022)
Supplement: Supplementary file 1 [file nanomaterials-07-00022-s001.pdf]

Supplementary Materials: A Novel Heterostructure of BiOI Nanosheets Anchored onto MWCNTs with Excellent Visible-Light Photocatalytic Activity

Shijie Li, Shiwei Hu, Kaibing Xu, Wei Jiang, Jianshe Liu and Zhaohui Wang

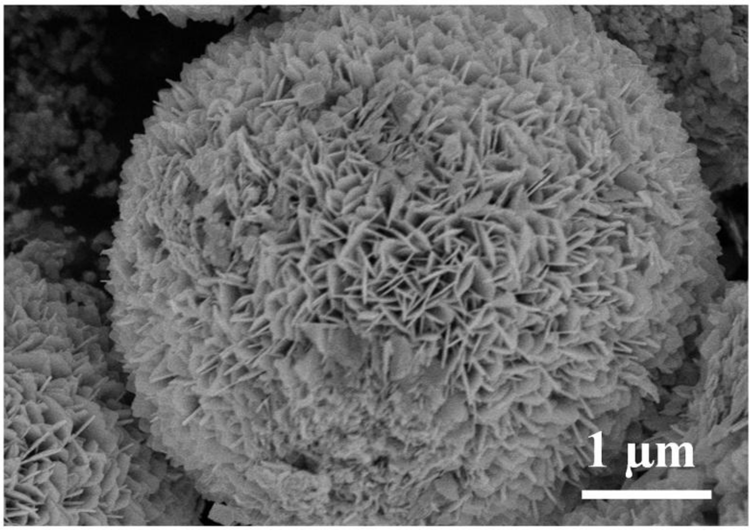

Figure S1. Scanning electron microscopy (SEM) image of pure BiOI.

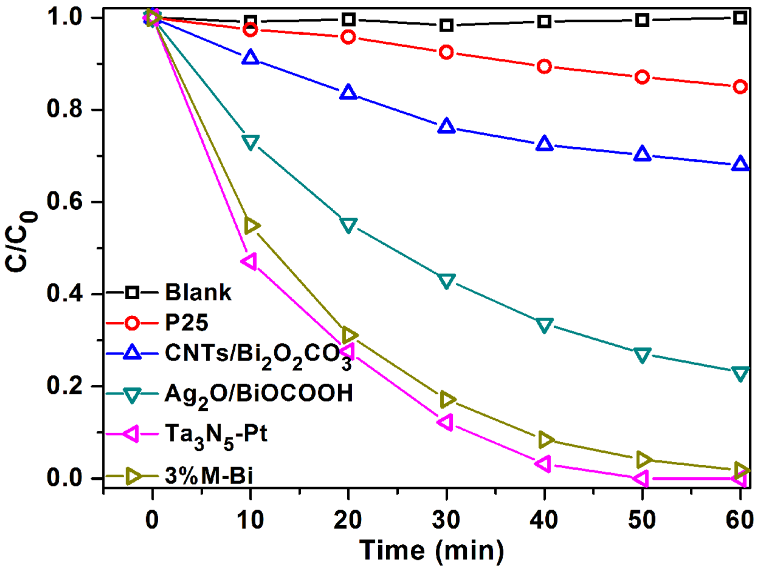

Figure S2. The photocatalytic degradation of RhB over P25, CNTs/$Bi_2O_2CO_3$, Ag_2O/BiOCOOH, Ta_3N_5-Pt, and 3%M-Bi (10 mg).

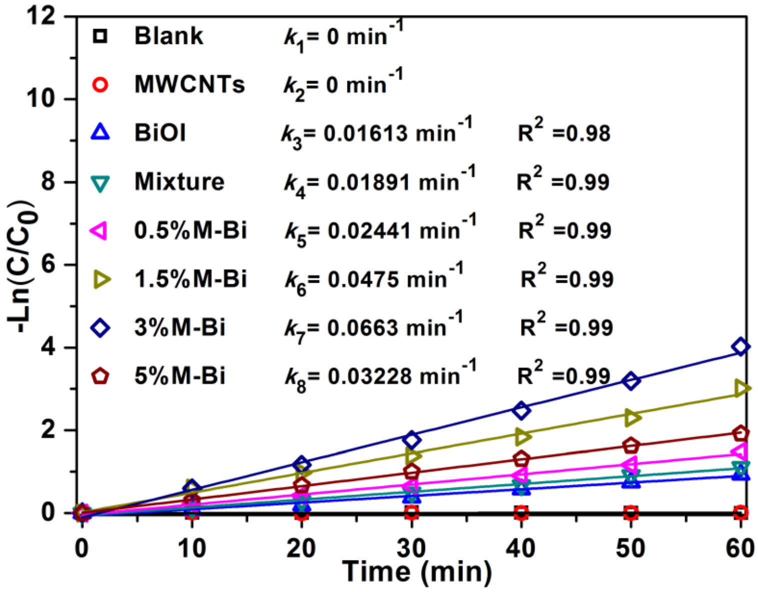

Figure S3. The rate constants of rhodamine B (RhB) in aqueous solution under visible-light irradiation (λ > 400 nm), in the absence of catalyst, and in the presence of different catalysts (10 mg).

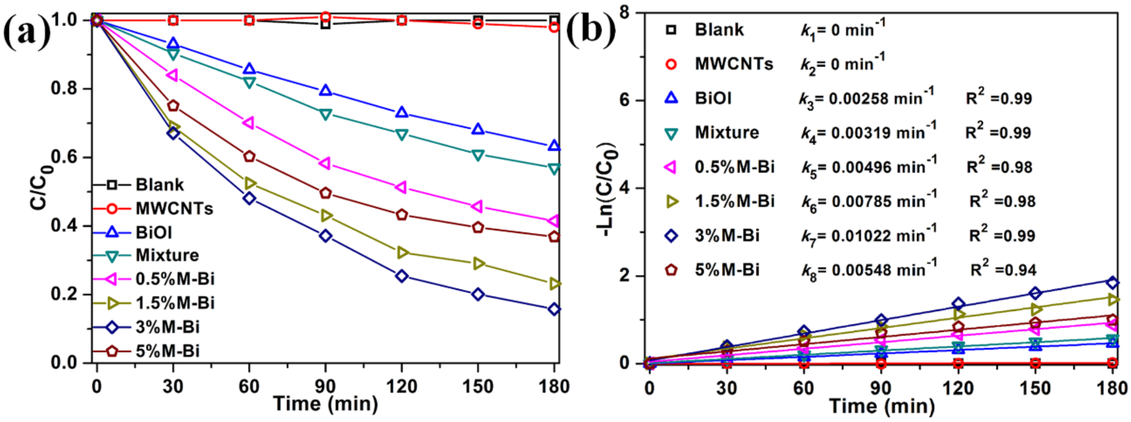

Figure S4. The degradation efficiencies (**a**) and rate constants (**b**) of methyl orange (MO) in aqueous solution (5 mg·L^{-1}, 50 mL) versus the exposure time under visible-light irradiation (λ > 400 nm), in the absence of catalysts and in the presence of as-prepared catalysts (10 mg).
